# The effect of self-determination interventions on reading outcomes for middle and high school students with learning disabilities: A systematic review

**DOI:** 10.1371/journal.pone.0362550

**Published:** 2026-11-12

**Authors:** Mohammed A. Aljufayr, Abdulrahman S. Alsultan, Abdulaziz M. Alasmari

**Affiliations:** 1 Department of Special Education, College of Education, Prince Sattam Bin Abdulaziz University, Al-Kharj, Saudi Arabia; 2 Department of Psychological and Behavioral Sciences, College of Arts and Humanities, Jazan University, Jizan, Saudi Arabia; University of Oviedo, SPAIN

## Abstract

**Background:**

Students with learning disabilities (LD) often struggle with reading skills, increasing the need for interventions that enhance their reading outcomes. Self-determination (SD) interventions can develop reading skills for students in all grade levels, but previous reviews of SD intervention studies have focused on elementary school students. This systematic review investigated the effects of SD interventions on reading outcomes for middle and high school students with LD.

**Methods:**

Seventeenth studies published between 1982 and 2025 in scholarly journals were reviewed, examining participant demographics, settings, designs, intervention procedures, dependent variables, interobserver agreement, procedural fidelity, social validity, maintenance, generalization, intervention effects, and quality.

**Results:**

The findings indicated that reading outcomes for middle and high school students with LD improved when using SD interventions. Limitations, implications for practice, and recommendations for future research are also discussed.

**Conclusions:**

The findings suggest that SD interventions represent practical, low-cost, and easily implemented strategies for improving reading outcomes among secondary students with LD; however, increased professional development and systematic teacher training are essential to support effective and widespread implementation in school settings.

## Introduction

Reading is a foundational and vital academic skill [1]. It is a crucial skill for acquiring knowledge [2]. Reading is a process of learning that can hugely contribute to mind development. Also, reading skills play a significant role in students’ self-improvement and success in life [3]. Failure to develop reading skills in early grades is likely to harm students’ learning in later grades, and may lead to negative consequences such as school dropout and behavioral problems [4]. Students with LD at all grade levels often struggle with reading skills. LD refers to neurodevelopmental disorders typically identified during the early developmental period in school, which appear in at least one of three academic areas: reading, writing, or math [5]. Students with LD represent the highest percentage (about 33%) of those who receive special education services [6]. According to De Paoli et al. [7], around 85% of students with LD exhibit a primary deficit in reading skills. Even though most students with LD are taught in general education settings with nondisabled students for approximately 80% of the school day, their performance in reading continues to be poor compared to their peers without disabilities [8].

When students experience continuous failure in reading during elementary school, it may negatively impact their motivation to read [9]. Middle and high school are also more challenging for students lacking foundational reading skills [8]. According to the National Joint Committee on Learning Disabilities [10], many middle and high school students with LD exhibit poor reading skills, with reading performance approximately five grade levels below their peers without LD. Therefore, there is a great need for interventions for struggling middle and high school readers that push them forward and promote their reading skills to increase their academic success.

One of the effective approaches to reading intervention is emphasizing self-determination (SD). Shogren et al. [11, p. 258] defined SD as follows:

[A] dispositional characteristic manifested as acting as the causal agent in one’s life. Self determined people (i.e., causal agents) act in service to freely chosen goals. Self-determined actions function to enable a person to be the causal agent in his or her life.

According to the literature [11–14], SD skills include (a) decision making, (b) choice making, (c) goal setting and attainment, (d) positive attribution of efficacy and expectancy, (e) self-awareness, (f) self-advocacy, (g) problem solving, (h) internal locus of control, (i) self-knowledge, and (j) self-management (i.e., self-monitoring, self-instruction, self-questioning, self-reinforcement, and self-evaluation). Individuals develop their SD skills from childhood through adulthood [15]. SD skills play an essential role in reading performance and school success [16]. Several previous studies have demonstrated positive effects of SD skills on reading outcomes for students with LD in different grade levels elementary [17] middle [18], and high [19].

Previous literature reviews about SD skills in education have focused on different variables. Some have targeted specific populations, such as students with intellectual disabilities [e.g., 20,21], behavior challenges [e.g., 22], attention-deficit/hyperactivity disorder [ADHD; e.g., 23], and LD [e.g., 12,24]. Other reviews focused on different dependent variables, such as behavior skills [e.g., 22], reading outcomes [e.g., 25,26], social skills [27], and academic skills [12]. Others examined individual components of SD, including self-monitoring [e.g., 26], goal setting [e.g., 28], self-advocacy [e.g., 29], and choice-making [e.g., 30]. While these reviews all examined the effects of SD interventions, they largely focused on elementary school students.

For example, Konrad et al. [12] specifically reviewed the impact of SD interventions on academic outcomes for students with LD and/or ADHD. While this review included 34 studies and indicated positive effects of these interventions, most participants were elementary school students, the results for academic outcomes were aggregated, and the focus was on all academic skills, making it complicated to conclude the interventions’ impacts on reading outcomes for middle and high school students. More recently, Didion et al. [24] reviewed 12 studies to examine the effects of SD interventions on reading performance for elementary school students with or at risk for LD. This review described how SD skills were taught and their effects on reading performance. However, the results cannot be generalized to students with LD in middle and high school, given that it focused on elementary school.

No previous literature review has specifically focused on how SD interventions affect middle and high school students with LD. Therefore, the current review aimed to provide more precise conclusions regarding these interventions’ impact on students in these grade levels. This review addressed the following research questions: First, what are the characteristics of SD studies regarding participant demographics, implementers, settings, reading outcomes, experimental designs, interobserver agreement, procedural fidelity, and social validity? Second, what are the effects of SD interventions on reading outcomes for middle and high school students with LD, and to what degree do the participants maintain and generalize reading skills? Third, what components of SD skills are used to enhance reading outcomes for students with LD? Fourth, what are the session frequency, length, and total duration of interventions in SD studies? Fifth, to what extent do studies examining SD interventions for middle and high school students with LD demonstrate rigor and quality?

## Methods

### Selection criteria

We included studies that met the following criteria. First, studies were published in English peer-reviewed journals. Second, studies employed an experimental design (i.e., single-case or group design) or a quasi-experimental design. Third, the intervention included one or more SD components: that is, a SD intervention was the main component or part of a multicomponent intervention. Fourth, studies included at least one dependent variable measuring reading outcomes, defined as any tasks directly related to reading skills (e.g., comprehension, fluency, or vocabulary). Studies measuring academic engagement or on-task behavior skills during reading instruction (e.g., looking at the texts, following along with other students) were excluded because they did not directly measure reading skills. Fifth, studies included at least one participant with LD. Sixth, Studies were included if at least one participant was identified by the authors as being in middle or high school. Given international variation in grade configurations, school level was defined according to authors’ classifications rather than a fixed grade range, with Grades 6–12 typically considered middle or high school unless otherwise specified.

We excluded studies not published in refereed journals (e.g., theses/dissertations), conceptual articles, practitioner papers, book chapters, and qualitative studies. Also, we excluded studies that did not present their data graphically or allow replication of treatment effects (e.g., AB, ABA) for single-case designs. For group designs, we excluded studies if the results were not disaggregated for participants with LD in middle or high schools.

### Search procedure

To identify studies, we developed the following search string of key terms: (“self -determin*” OR “self-monitor*” OR “choice mak*” OR “problem solv*” OR “goal set*” OR “decision mak*” OR “self-regulat*” OR “self-advoc*” OR “self-awar*” OR “self-efficacy” OR “self-instruct*” OR “self-evaluat*” OR “self-reinforce*” OR attain* OR “self-manage*” OR “self-observ*” OR “self-knowledge” OR attribution OR “internal locus of control” OR “self-recruit*” OR “self-question*” OR “self-graph*” OR “self-record*”) AND (“learning dis*” OR “language dis*” OR “special education” OR “disabilit*” OR dyslexi* OR “learning diff*” OR “reading dis*” OR “reading diff*”) AND (reading OR literacy OR phonic* OR “phonemic awareness” OR fluency OR comprehension OR vocabulary) AND (“middle school” OR “junior high” OR “adolescent” OR “high school” OR “secondary education” OR “secondary school” OR “grade 6” OR “grade 7” OR “grade 8” OR “grade 9” OR “grade 10” OR “grade 11” OR “grade 12” OR “6th grade*” OR “7th grade*” OR “8th grade*” OR “9th grade*” OR “10th grade*” OR “11th grade*” OR “12th grade*” OR “sixth grade*” OR “seventh grade*” OR “eighth grade*” OR “ninth grade*” OR “tenth grade*” OR “eleventh grade*” OR “twelfth grade*”).

We conducted an electronic search on December 21^st^, 2025, through The Ohio State University Library search interface using six databases; APA PsycINFO, ERIC, Academic Search Complete, Academic Search Premier, Education Full Text, and Education Research Complete databases. The databases were all searched on the same date. Also, the same search syntax and search limits were applied across all six databases. The complete search strategy is provided in the Supporting information (S1 Table). We limited the search to peer-reviewed English articles and found 2,203 results. This search returned 1,065 hits after duplicates were automatically removed through The Ohio State University Library search system. The 1,065 articles were subsequently exported to Microsoft Excel for record management and screening. Reviewing each study’s title and abstract narrowed the list to 177 potential studies. After reading the full text of these articles, we identified 12 studies that met the inclusion criteria. A backward search reviewing the references of the 12 included studies yielded only one additional article, but a forward search of the 12 studies using Google Scholar identified four additional studies. Finally, we conducted a hand search of the following journals: *Remedial and Special Education, Exceptional Children, Journal of Learning Disabilities, Learning Disability Quarterly,* and *Learning Disabilities Research and Practice.* However, we did not find additional studies. Thus, a total of 17 studies met the inclusion criteria. The PRISMA flow diagram (Fig 1) shows the study search procedures.

**Fig 1:**
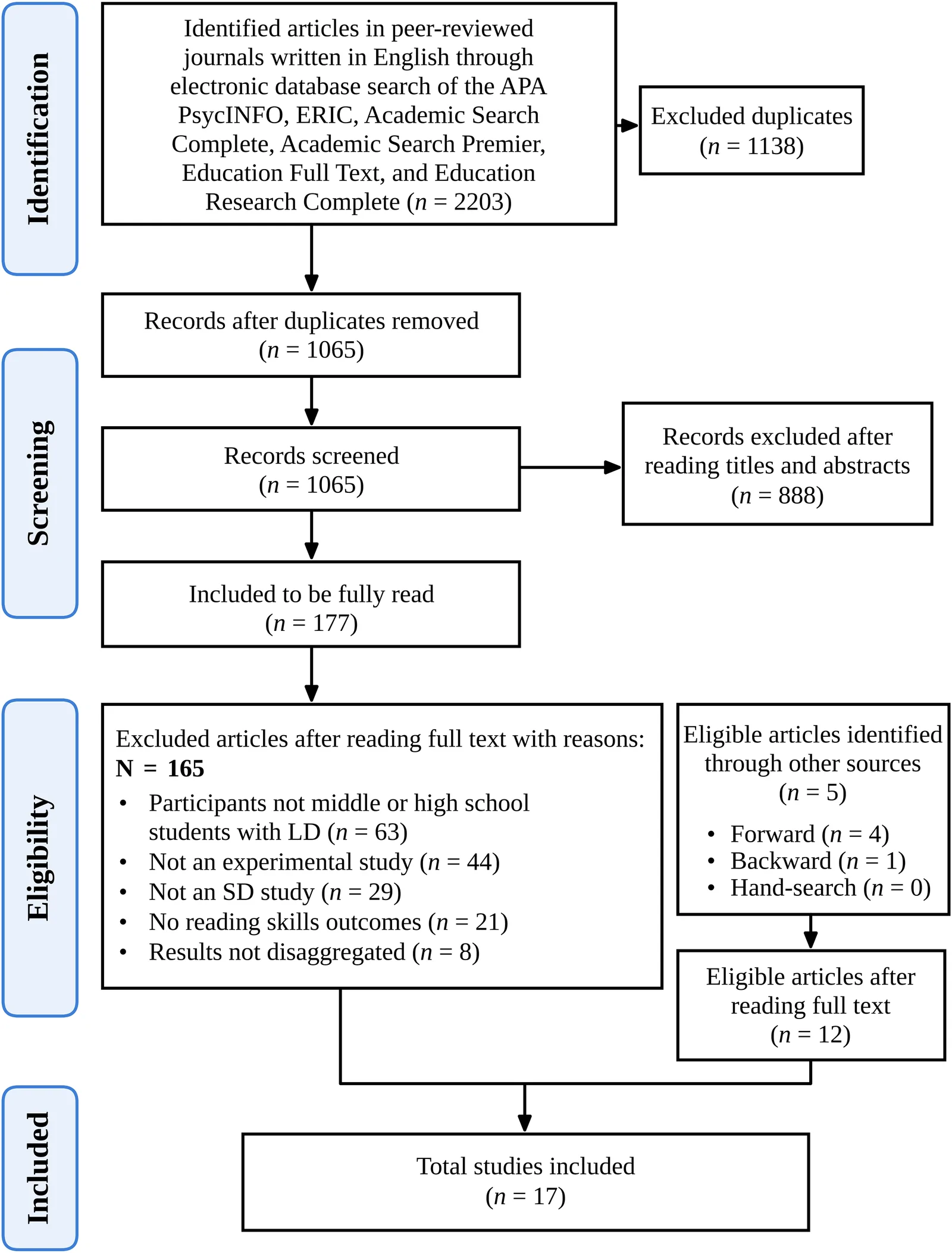
PRISMA flow diagram of study search procedures.

This systematic review was not prospectively registered in PROSPERO, the Open Science Framework, the Campbell Collaboration, or another systematic review registry. Also, no formal protocol was prepared or registered prior to the review launch. The PRISMA checklist was used to guide the reporting of this systematic review (see S1 File).

### Coding procedures

#### Student demographics.

We coded the disability status of participants as LD alone or LD with other disabilities. Grade level was coded as middle school (Grades 6–8), high school (Grades 9–12), or unclear (i.e., authors indicated that participants were middle or high school students without specifying the exact grade level[s]). We coded age as the mean, range, or not reported; gender as male, female, or not reported; and race/ethnicity as Caucasian/White, Black/African American, Asian American, Hispanic/Latino, Native Hawaiian/Pacific Islander, Turkish, Biracial, other races, or not reported.

#### Implementer roles and settings.

The implementer of the intervention in each study was coded as author/researcher, general education teacher, special education teacher, graduate student, undergraduate student, or not reported. Also, we coded whether training pertaining to the study intervention was provided to the implementer. Settings were coded as general education classroom (including inclusive classrooms), special education classroom, resource room, reading clinic, other school settings (e.g., quiet rooms outside the classroom, staff meeting room), or not reported.

#### Study designs.

We coded single-case designs as multiple-baseline/probe designs across participants, behaviors, or contexts/stimulus conditions (e.g., settings, materials); reversal; alternating treatments; changing criteria; or a combination of these designs. If a study had a design nested within another design (e.g., a combined alternating-treatments design and multiple-baseline design), we coded all included designs. Group design studies were coded as experimental or quasi-experimental designs. Group experimental designs included a pretest–posttest randomized experimental design with treatment comparison, a posttest randomized experimental design with treatment comparison, and repeated measures of posttests.

#### Intervention procedures.

We coded whether the study included one or more SD components, including goal setting (i.e., teaching students to set goals and recognize steps required to complete them; [14] and self-management, which encompassed self-monitoring, self-questioning, self-evaluation, self-reinforcement, and self-instruction. Self-monitoring (i.e., teaching students to observe or record their own performance and whether they performed a target behavior or met target criteria. This could involve using sheets to record questions or answers, graphs to record their performance, prompt cards or checklists to check off strategy steps when applying them, or cue cards to remember paraphrase requirements or criteria of the strategy). Self-questioning (i.e., teaching students to ask themselves questions about a text, whether student-generated or teacher-generated questions). Self-evaluation (i.e., teaching students to progress their performance over time by comparing it with a desired goal). Self-reinforcement (i.e., teaching students to reward themselves after accomplishing targeted goals). Lastly, self-instruction (i.e., teaching students to use verbal prompts that guide problem solving and support them during challenging academic tasks [14,24,26,31–33].

Additionally, some studies used these SD components as part of multicomponent instructional strategies. These strategies include the Think Before Reading, Think While Reading, Think After Reading (TWA), a strategy that instructs students to think about a passage and ask themselves questions before, during, and after reading [34]. The Self-Regulated Strategy Development (SRSD) model included six stages: developing and activating background knowledge, discussing the strategy, modeling the strategy, memorizing the strategy, supporting student, and independent performance [35]. The Read–Ask–Put (RAP) strategy involved three steps: students read a paragraph, ask themselves about the main idea of the paragraph and provide two supporting details, and paraphrase it. Questions are written on index cards for future use [36]. The Think–Read–Ask–Put (TRAP) strategy was similar to the RAP strategy, but added a step at the beginning in which students ask themselves why they are reading and what they already know about the topic [37]. The summarization strategy involved students asking themselves after reading each paragraph, “Who or what is the paragraph about? What is happening to them?” and then using the answers to provide a summary sentence [38]. The Survey, Question, Read, Recite, Review (SQ3R) strategy consisted of five steps: survey (read the title and the subheads to get an idea about the passage), question (turn the title and the subheads into questions), read the passage, recite (retell the important details in each subhead), and review (read each subhead and review the important details you retold) [39].

Several additional strategies were built on these approaches. The Embedded Story Structure (ESS) strategy involved self-questioning, story-structure analysis, and summary writing, whereas Comprehension Skill Instruction (CSI) incorporated the LINCS Vocabulary Strategy, Question-Answer Relationships, and Semantic Summary Mapping [40–42]. Another summarization strategy was “Identify important details, Delete trivial details, Eliminate redundant details, Add a term for a list of words or concepts, and Summarize (IDEAS)”, which consisted of five instructional steps aligned with its name [37, pp. 54–55]. Finally, the “Write the main idea, Identify important information, Number the pieces of identified information, Develop sentences, Organize sentences using transition words, and Write an ending sentence (WINDOW)” strategy consisted of six structured steps to support written summarization [43, p. 6].

#### Dependent variables.

The measures of dependent variables (reading outcomes) were coded, including accuracy (i.e., number or percentage of comprehension questions answered correctly, correct identification of main ideas, accurate summaries, and correct paraphrases of key details), recall accuracy (i.e., percentage or amount of information recalled/retold from a passage correctly), and productivity (i.e., percentage or number of of reading comprehension questions completed).

Also, types of outcome measures were coded as published standardized measures or researcher-developed measures. Published standardized measures were standardized tests that measured reading outcomes, such as the Nelson Reading Test [44] and Stanford Diagnostic Reading Test [45]. Researcher-developed measures were specific measures designed for a specific study.

#### Interobserver agreement and procedural fidelity.

The mean interobserver agreement (IOA) and procedural fidelity (PF) were coded for each study. If multiple means of IOA or PF were reported, the lowest mean was coded for both. Also, we coded whether the mean IOA and PF were 80% or more and whether IOA and PF were examined in 20% of sessions at minimum; these levels were chosen because they have been used as minimal standards [46].

#### Social validity.

We coded whether the social validity of the intervention was measured and with whom, including direct consumers (students) or indirect consumers (teachers or parents; [47]). Also, the method used to measure social validity was coded as survey/questionnaire, interview, or observation.

#### Maintenance and generalization.

We coded whether maintenance was measured in each study. If maintenance was measured, we coded the period of measurement as 0–2 weeks, 2–4 weeks, 4–6 weeks, 6–12 weeks, greater than 3 months, or unclear (i.e., the maintenance time was not described). Also, we coded whether the generalization of skills was measured across settings, times, activities, materials/texts, or people. Finally, data were collected on whether maintenance and generalization data were visually presented in the single-case design studies.

#### Intervention intensity.

For the single-case design studies, we coded the frequency of sessions (per day/week), session duration, and total study duration. For the group design studies, we coded the number of training/instruction days, session duration, total instruction duration, and total study length.

#### Efficacy of interventions.

For the single-case design studies, we used success estimates to examine experimental effects [48]. Based on visual analysis of the data in graphs, success estimates summarize the number of times an effect was demonstrated and the number of opportunities to demonstrate an effect. While other methods exist to analyze effects in single-case studies (e.g., percentage of non-overlapping data, percentage of all non-overlapping data, percentage of data exceeding the median), they have some limitations in quantitatively analyzing the graphs of all designs in single-case research [49]. Thus, we decided to use success estimates because they summarize the visual analysis, the most established approach for analyzing single-case design research [46]. To calculate success estimates, we visually analyzed each graph and recorded, based on the experimental design, the number of opportunities to demonstrate an effect and the number of effects identified. Also, level, trend, variability within conditions, overlap between conditions, and immediacy of effects were visually analyzed to determine whether there was an effect or no effect [46,50]. For some studies, we calculated success estimates for only participants who met the inclusion criteria.

In addition, we identified one single-case design study with an alternating treatments design, which included three interventions. For this study, six success estimates were calculated; the first three success estimates were derived by independently analyzing the effect of each intervention. The other three success estimates assessed how distinct the outcomes were between the interventions—that is, the degree of separation in effectiveness among the independent variables [51]. For all single-case studies, success estimates for maintenance and generalization were calculated separately from the general experimental data. To do that, we counted the change from intervention to maintenance or generalization as an opportunity to demonstrate an effect. A maintenance effect was counted if the response remained at the same levels recorded during the intervention over time; a generalization effect was counted if the participant could respond to new settings, times, activities, or materials/texts at the same levels as during the intervention [51].

We used Cohen’s *d* to summarize the effects of the group design studies. The effect size was calculated using standardized mean effect sizes, in which the difference between the posttest means for the treatment and control groups was divided by a pooled standard deviation [52]. A control group was defined as a group in which no treatment was provided or no SD components were included. However, the control group could also be considered a treatment group if the study included only two treatment groups and the author considered one as a control group. For a study comparing three groups, in which there were two treatment groups receiving SD interventions and one control group, we calculated Cohen’s *d* by comparing each treatment group to the control group to examine which group had a higher effect. Also, we calculated Cohen’s *d* by comparing the first treatment effect to the second treatment effect to examine which treatment had a higher effect. If the posttest mean and standard deviations were not reported in the study, and the information needed to calculate the effect size was not reported, the effect size was not coded. Effect sizes were categorized as follows: 0.20 = “small,” 0.50 = “medium,” and 0.80 = “large,” as recommended in Cohen’s [53] scale.

#### Quality of studies.

The methodical quality of the studies was assessed using the quality indicators (QIs) proposed and explained by the Council for Exceptional Children (CEC) for single-case and group design studies [54]. The QIs include items in eight categories: context and setting, participants, intervention agent, description of practice, implementation fidelity, internal validity, outcome measures/dependent variable, and data analysis. Of these items, 22 apply to single-case designs and 24 to group studies; most of the QIs apply to both types, with slight variations in some items. Based on the descriptions of the eight QI categories, we determined whether each study provided sufficient information to meet each QI. Each item was scored as 0 (not met) or 1 (met), and the total for each study was calculated out of 22 for the single-case design studies and 24 for the group design studies.

#### Risk of bias assessment.

We assessed the risk of bias using design-specific tools. For the 12 single-case design studies, we used the Single-Case Design Risk of Bias tool (RoB) [55], whereas the five group-design studies were evaluated using the revised Cochrane Risk of Bias 2 tool (RoB 2) [56]. The RoB assesses potential bias across selection, performance, detection, and other sources of bias, with domains rated as low, high, or unclear risk of bias. RoB 2 assesses bias across the randomization process, deviations from intended interventions, missing outcome data, measurement of the outcome, and selection of the reported result, with domains rated as low risk, some concerns, or high risk of bias. We used design-specific tools to ensure that the methodological features of each study design were appropriately assessed.

#### Reliability of coding.

To ensure the reliability of variable coding, success estimates, QI assessment, and risk of bias assessment, the second author was engaged. The first author trained the second author on the coding manual, practicing coding procedures, determining success estimates, assessing the quality of the studies, and risk of bias assessment. Training required achieving at least 90% agreement on a practice data set before independent coding began. Following this benchmark, both authors independently coded the data.

Intercoder reliability (ICR) for the search strategy was assessed during the initial screening phase, which involved evaluating titles and abstracts for 269 of the 1,065 unduplicated results (25.2%). We used a point-by-point agreement method (i.e., dividing the number of agreements by the total number of agreements plus disagreements and multiplying by 100). The mean intercoder agreement was 93.3%, with discrepancies resolved through consensus. Second, ICR was assessed for 54 of the 177 full-text studies reviewed (30.5%), using the point-by-point agreement method. This stage yielded a mean agreement of 94.4%, and all discrepancies were resolved through consensus. Third, during the backward, forward (via Google Scholar), and hand search processes, both coders independently identified the same five additional studies, achieving 100% agreement.

ICR was calculated separately for single-case and group-design studies due to differences in coding frameworks, study effects, quality indicators, and risk of bias assessment. For single-case studies, agreement was calculated across all coded variables, including study characteristics, success estimates, and single-case quality indicators. ICR was assessed for 4 of the 12 single-case studies (33.3%), using the point-by-point agreement method. This stage yielded a mean agreement of 91.8%, and all discrepancies were resolved through consensus. For group-design studies, agreement was calculated across study characteristics, standardized effect sizes, and group-design quality indicators. ICR was assessed for 2 of the 5 group-design studies (40%), using the point-by-point agreement method. This stage yielded a mean agreement of 93.6%, and all discrepancies were resolved through consensus. The list of included and excluded studies with intercoder reliability are provided in the Supporting information (see S2 Table and S3 Table).

## Results

Seventeen studies, comprising 19 experiments, met the inclusion criteria for this review. Findings or the coded variables, along with summaries of the intervention procedures and dependent variables, are presented and synthesized below and presented in Tables 1–5.

**Table 1:** Implementer, setting, and intervention intensity by study.

Intervention intensity					
Study	Implementer	Setting	Sessions per day/week	Session duration	Study length
Berkeley et al. [57]	Special + general education teachers	General classrooms	(1 day)	1 hr	3 days
Clark et al. [58]	Special education teachers	Area outside the classroom	5	NR	NR
Crabtree et al. [19]	Author/researcher	Resource room	3	15-30 min	NR
Faggella-Luby et al. [59]	Author/researcher + general education teacher	General classroom	(9 days)	1.5-2 hr (total = 17 hr)	NR
Firat [60]	NR	NR	2	NR	8 weeks
Hagaman et al. [18]	Author/researcher + undergraduate student	Quiet rooms outside the classroom	3	45 min	NR
Jitendra et al. [61]	Graduate student	Resource room	NR	40-50 min	3 months
Lauterbach & Bender [62]	NR	NR	NR	NR	NR
Malone & Mastropieri [38]	Author/researcher	Quiet rooms outside the classroom	(2 days)	NR	3 days
Manset-Williamson et al. [63]	NR	Reading clinic	4	NR	6 weeks
McCormick & Cooper [64]	Graduate student	Unused classroom & staff meeting room	5	NR	**EX1** and **EX3**: 4.5 weeks; **EX2**: 6 weeks
Pitzel et al. [65]	Author/researcher	Multipurpose room	NR	NR	NR
Saddler et al. [43]	Author/researcher	General classroom	3-4	30 min	NR
Sanders et al. [66]	Author/researcher	A separate classroom	NR	25-45 min	NR
Söğüt & Melekoğlu [67]	General education teachers	Resource room	UC	15 to 30 min	3 weeks
Shimabukuro et al. [68]	Special + general education teachers	Special education classroom	NR	45 min	NR
Wong & Jones [33]	Author/researcher	NR	(5 days)	1-2 hr (total = 7 hr)	9 days

*Note*. EX = experiment; NR = not reported. Numbers in parentheses represent training/instruction days in group design studies; UC: Unclear.

**Table 2:** Designs, intervention components, dependent variables, and success estimates of single-case design studies (n = 12).

					Success estimates					
Intervention components					IN		M		G	
Study	Design	SD components	Independent variables	Dependent variables (type of DM)	X/Y	%	X/Y	%	X/Y	%
Clark et al. [58]	Multiple-baseline design across materials	**Self-management** • Self-questioning	Students read a passage and used the visual imagery strategy (make and describe visual images representing the passage and assess these images to see if they can help them with reading comprehension). After reaching the criterion of this strategy, students were taught to read a passage, self-generate “WH” questions about the text, ask themselves, and answer during reading.	• % of correct responses to comprehension questions on grade level (**accuracy**) • % of correct responses to comprehension questions on ability level (**accuracy**)	1/1 1/1	100 100				
Crabtree et al. [19]	Multiple-baseline design across participants	**Self-management** • Self-questioning • Self-monitoring	Students read a story, used prompt marks to stop at three predetermined times during reading to ask themselves questions generated by the teacher (e.g., who are the main characters?), and then recorded their answers in the self-monitoring sheet in specific boxes.	• Number of short answer comprehension questions answered correctly (**accuracy**) • Number of story facts recalled correctly (**recall accuracy**)	3/3 3/3	100 100	3/3 3/3	100 100		
Firat [60]	Multiple-probe design across participants	**Goal setting** **Self-management** • Self-questioning • Self-monitoring • Self-reinforcement • Self-instruction	Students implemented the nine steps of the TWA strategy during reading using the SRSD model. They set goals to implement the TWA strategy steps at the beginning of their reading, asked themselves questions embedded through the TWA strategy (e.g., main idea), checked their use of the strategy steps using a checklist, and finally rewarded themselves after completing the strategy steps by painting a portion of a rocket after each session.	• Number of comprehension questions answered correctly (**accuracy)**	3/3	100	3/3	100	UN	
Hagaman et al. [18]	Multiple-baseline design across participants	**Goal setting** **Self-management** • Self-questioning • Self-monitoring • Self-instruction • Self-evaluation	Students implemented the steps of the TRAP strategy during reading using the SRSD model. They were shown a graph representing their performance in the baseline and asked to set a goal of how much information can be retold when using the TRAP strategy. Also, they asked themselves questions embedded through the TRAP strategy (e.g., main idea and details), monitored their strategy use by writing “TRAP” in their paper, checked off strategies’ steps, and self-graphed the percentage of text recalled. Finally, they evaluated the progress of their performance over time toward their desired goal.	• % of text recalled on main idea and details (**recall accuracy**) • Number of short answer comprehension questions answered correctly (**accuracy)**	1/1 1/1	100 100	1/1 1/1	100 100		
Jitendra et al. [61]	Multiple-baseline design across participants	**Self-management** • Self-monitoring	Students read passages and used prompt cards containing steps for identifying the main idea. They checked off these steps to find the main idea and summarize the passages.	• % of questions answered correctly on narrative passages (**accuracy**) • % of questions answered correctly on expository passages **(accuracy)**	2/3 2/3	66.6 66.6	1/3 1/3	33.3 33.3	UN UN	
Lauterbach & Bender [62]	Multiple-baseline design across participant + changing criteria	**Self-management** • Self-questioning • Self-monitoring	Students implemented the steps of the RAP strategy while reading passages. They asked themselves about the main idea, provided two supporting details, and paraphrased each paragraph. In addition, they used cue cards with the requirements of a correct paraphrase to check them while applying the RAP strategy.	• % of correct paraphrasing of main ideas and supporting details (**accuracy**) • % of comprehension questions, on main idea and supporting details, answered correctly (**accuracy**)	8/8 6/8	100 75			2/2	100
Manset-Williamson et al. [63]	Multiple-baseline design across participant	**Self-management** • Self-questioning • Self-monitoring	Students used a self-monitoring strategy called FIST while reading a passage. They read the first sentence, then used the embedded prompt (FI) as a reminder to generate a question for each paragraph (e.g., “What did the freighter do?”) and another prompt (ST) at the end to indicate whether their questions were answered. They used voice notes to record questions and summarize responses.	• Number of inference questions, representing main idea, answered correctly (**accuracy**) • Number of ideas expressed during retelling (**recall accuracy**)	1/2 1/2	50 50				
McCormick & Cooper [64]	**EX1:** Multiple-baseline design across participants	**EX1**: **Self-management** • Self-questioning	Before reading passages, students were provided with three words on flashcards, considered to be difficult for students, and taught to pronounce and define these words correctly. During reading passages (around 1,000 words for 10 min), students implemented the SQ3R strategy, including turning the title and the subheads into questions. Students earned tokens for information retold, then exchanged them for items (e.g., candies, drinks).	• % of retelling information on long review passages (**recall accuracy**)	2/9	22.2				
	**EX2:** Alternating treatments design	**EX2**: **Self-management** • Self-questioning • Self-monitoring	Same as EX1, except: (a) no word preview, (b) no token economy, instead, self-graphing of the number of information retold, (c) three conditions were used: SQ3R long review (passages were around 1,000 words to read for 10 min), short/review (300 words and 4 min), and short prompt (same as short/review except that prompts were provided by the teacher during the recite period for the occurrence of information units).	• % of retelling information on long review passages + short review passages + short prompt (**recall accuracy**)	18/30 **SP** = 14/30	60 46.7				
	**EX3:** Multiple-baseline design across participant + reversal design	**EX3**: **Self-management** • Self-questioning • Self-monitoring	Same as EX1, except: (a) no token economy, instead, self-graphing of the number of information retold, (b) passages were around 300 words to read for 4 min (short review).	• % of retelling information on short review passages (**recall accuracy**)	3/12	25				
Pitzel et al. [65]	Multiple-baseline design across participant	**Goal setting** **Self-management** • Self-monitoring • Self-instruction • Self-questioning • Self-reinforcement	Students implemented the steps of the TRAP+IDEAS strategies during reading within the SRSD model. They were instructed to set a reading goal and monitor their progress. Students independently followed the strategy steps as part of self-instruction and engaged in self-questioning embedded in the TRAP strategy (e.g., main idea and details). Finally, students self-reinforced their performance.	• Number of main ideas and details included within the written summary (**accuracy)**	2/3	66.6	2/2	100		
Saddler et al. [43]	Multiple-baseline design across participant	**Goal setting** **Self-management** • Self-monitoring • Self-instruction • Self-evaluation • Self-reinforcement	Students implemented the steps of the WINDOW strategy during reading within the SRSD model. Students were instructed to set a reading goal and monitor their progress using a Rocket Chart. They also used a self-check guide as a part of self-instruction. Finally, students self-evaluated and reinforced their performance.	• Number of main ideas and details included within the written summary (**accuracy)**	3/3	100				
Sanders et al. [66]	Multiple-baseline design across participant	**Goal setting** **Self-management** • Self-monitoring • Self-instruction • Self-questioning • Self-reinforcement	Students implemented the steps of the TRAP strategy during reading within the SRSD model. They were instructed to set a reading goal and graph their performance. They monitored their progress using a checklist, employed self-statements as a part of self-instruction, and engaged in self-questioning embedded in the TRAP strategy (e.g., main idea and details). Finally, they reinforced their performance through positive self-praise and by selecting a brief preferred activity.	• Number of main ideas and details identified (**accuracy)**	0/1	0			1/1	100
Shimabukuro et al. [68]	Multiple-baseline design across materials	**Self-management** • Self-monitoring	Students were taught to correct answers to their reading comprehension questions, then to calculate and record on a sheet the number of questions completed (productivity) or answered correctly (accuracy) and self-graph their reading accuracy and productivity level in answering these questions.	• % of questions answered correctly (**accuracy**) • % of questions completed relative to the number of questions assigned (**productivity**)	3/3 3/3	100 100				

Note. DM = dependent measures; EX = experiment; G = generalization; IN = intervention; M = maintenance; SD = self-determination; SP = separation effect in the alternating treatments design; SRSD = Self-Regulated Strategy Development; UN = unable to calculate.

**Table 3:** Designs, intervention components, dependent variables, and effect sizes of group design studies (n = 5).

Intervention components					
Study	Design	SD components	Independent variables	Dependent variables (type of DM)	Effect size *d*
Berkeley et al. [57]	Pretest–posttest with treatment comparison	**Self-management** • SQ • Self-monitoring	**CO:** Students read passages containing headings and subheadings and answered questions at the end. **T:** Students were taught to implement SQ strategy steps. Students generated questions at the beginning of each paragraph by turning the headings and subheadings into questions, read the section, tried to answer their questions, and then revised the passage to check understanding or ask the teacher questions. Additionally, students used monitoring sheets to record their questions, then after reading each section, indicated whether they successfully answered them by circling “yes” or “no” on the monitoring sheet.	• Number of comprehension questions answered correctly (**accuracy**)	*Multiple-choice*: T < CO: *d* = −0.26 *Open-ended*: T > CO: *d* = 0.11
Faggella-Luby et al. [59]	Pretest–posttest with treatment comparison	**Self-management** • SQ • Self-monitoring	**CO**: Students were taught the CSI strategy, including three reading strategies, one being the QAR strategy. Students used a graphic organizer to implement the three strategies. **T**: Students were taught the ESS strategy, including three reading strategies, one being SQ. During reading a story, students asked themselves and answered seven types of “WH” questions generated by the teacher. Questions were related to essential components of the story structure (e.g., the main character, place, and time). A graphic organizer was used to implement the three strategies.	• Number of questions answered correctly of story information (**accuracy**)	T > CO: *d* = 0.93
Malone & Mastropieri [38]	Only posttest with treatment comparison	**Self-management** • SQ • Self-monitoring	Two treatment groups and one comparison group were included. **CO**: Students read stories, practiced difficult words, and answered story questions. **T1: Summarization strategy (S)**, which included SQ (more information about the strategy is explained in the Method section). **T2: Summarization strategy and self-monitoring (S + SM):** students used a prompt card containing the summarization steps (e.g., who, what, summary sentence) and checked them off when applying each step.	• % of texts recalled on short-answer questions (**recall accuracy**) • % of texts recalled on short-answer questions (**recall accuracy**) • % of texts recalled on short-answer questions (**recall accuracy**)	*Posttest of training:* T1 (S)> CO: *d* = 2.95 T2 (S + SM)> CO: *d* = 2.18 T2 (S + SM) > T1 (S): *d* = 0.37 *Near-transfer:* T1 (S)> CO: *d* = 1.35 T2 (S + SM)> CO: *d* = 1.48 T2 (S + SM) < T1 (S): *d* = −0.03 *Far-transfer:* T1 (S)> CO: *d* = 1.70 T2 (S + SM)> CO: *d* = 2.13 T2 (S + SM) > T1 (S): *d* = 0.77
Söğüt & Melekoğlu [67]	Pretest–posttest with treatment comparison	**Goal setting** **Self-management** • Self-monitoring • Self-instruction • Self-evaluation	**T1**: Students were taught using the Self- Determination Learning Model of Instruction (SDLMI). They were instructed to set a reading goal and monitor their progress. Students independently followed and implemented the steps of the strategy as part of self- instruction. Finally, they evaluated their performance. **T2 (Non-SDLMI):** Students were taught using modeling and guided practice without implementation of the SDLMI model. **CO (Business-As-Usual**): Students read stories without receiving any intervention.	• Standardized test scores reflecting the number of reading comprehension questions answered correctly (**accuracy)**	T1 (SDLMI)> CO: *d* = 1.82 T2 (Non-SDLMI)> CO: *d* = 0.13
Wong & Jones [33]	Only posttest (repeated measures) with treatment comparison	**Self-management** • SQ • Self-monitoring	Both conditions received training on the main idea concept. **CO**: Students read a passage, judged its quality, and got help with vocabulary or decoding problems. **T**: Students received SQ training, consisting of five steps to follow while reading a passage: (a) ask yourself, “What are you studying this passage for?”; (b) underline the main ideas; (c) generate questions about the main ideas; (d) answer your questions; (e) revise your questions and answers. Help was provided with vocabulary or decoding problems. Additionally, students used prompt cards that included the strategy steps and criteria for creating good questions and checked them off while applying the strategy.	• Number of open-ended comprehension questions answered correctly (**accuracy**) • Number of idea units recalled (**recall accuracy**)	UN UN

Note. CO = comparison group; CSI = Comprehension Skill Instruction; DM = dependent measures; ESS = Embedded Story Structure; QAR: Question-Answer Relationships strategy; SD = self-determination; SQ = self-questioning; T = treatment group; UN = unable to calculate.

**Table 4:** Quality indicators met by single-case design studies (n = 12).

	Quality indicators (22 items)																						
Study	1.1	2.1	2.2	3.1	3.2	4.1	4.2	5.1	5.2	5.3	6.1	6.2	6.3	6.5	6.6	6.7	7.1	7.2	7.3	7.4	7.5	8.2	Total
**Clark et al. [58]**	1	1	1	1	1	1	1	0	0	0	1	0	1	0	0	0	1	1	1	0	1	0	13
**Crabtree et al. [19]**	1	1	1	0	1	1	1	1	1	1	1	1	1	1	1	1	1	1	1	1	1	1	21
**Firat [60]**	0	1	0	0	1	1	1	1	1	1	1	0	0	1	1	1	1	0	1	1	1	1	16
**Hagaman et al. [18]**	1	1	1	1	1	1	1	1	1	0	1	1	1	1	1	1	1	1	1	1	1	1	21
**Jitendra et al. [61]**	1	1	1	1	0	1	1	1	1	0	1	0	0	0	1	1	1	1	1	1	1	1	17
**Lauterbach & Bender [62]**	0	0	1	0	0	1	1	0	0	0	1	1	1	1	0	1	1	1	1	1	1	1	14
**Manset-Williamson et al. [63]**	1	1	1	0	0	1	1	0	1	0	0	1	1	0	1	1	1	1	0	0	1	1	14
**McCormick & Cooper [64]**	1	1	1	1	1	1	1	1	1	1	1	1	1	1	1	1	1	1	1	1	1	1	22
**Pitzel et al. [65]**	1	1	1	1	1	1	1	1	1	1	1	1	1	0	1	1	1	1	1	1	1	0	20
**Saddler et al. [43]**	1	1	1	1	1	1	1	1	0	0	1	1	1	0	1	1	1	1	1	1	0	1	18
**Sanders et al. [66]**	0	1	1	1	1	1	1	1	1	1	1	1	1	0	1	1	1	1	1	1	1	1	20
**Shimabukuro et al. [68]**	1	1	1	0	0	1	1	0	0	0	1	0	0	1	1	1	1	1	1	1	1	1	15
**Total**	9	11	11	7	8	12	12	8	8	5	11	8	9	5	10	11	12	11	11	10	11	10	

*Note*. 1.1 Context/setting description; 2.1 Participant description; 2.2 Participant disability; 3.1 Role and description; 3.2 Training description; 4.1 Intervention procedures; 4.2 Materials description; 5.1 Assessing/reporting implementation fidelity; 5.2 Assessing/reporting fidelity dosage or exposure; 5.3; Assessing fidelity across relevant conditions/throughout study; 6.1 Independent variable (IV) systematically manipulated; 6.2 Baseline description; 6.3 No or limited access to IV during baseline; 6.5 Design provides at least three demonstrations of experimental effect at three different times; 6.6 Baseline phase contains at least three data points; 6.7 Design controls for common threats to internal validity; 7.1 Socially important outcomes; 7.2 Description of dependent variable measures; 7.3 Reports effects of the intervention on all measures; 7.4 Minimum of three data points per phase; 7.5 Adequate interobserver agreement; 8.2 Study provides single-case graph clearly representing outcome data across all study phases.

**Table 5:** Quality indicators met by group design studies (n = 5).

	Quality indicators (24 items)																								
Study	1.1	2.1	2.2	3.1	3.2	4.1	4.2	5.1	5.2	5.3	6.1	6.2	6.3	6.4	6.8	6.9	7.1	7.2	7.3	7.4	7.5	7.6	8.1	8.3	Total
**Berkeley et al. [57]**	1	1	0	1	1	1	1	1	1	1	1	1	1	1	1	1	1	1	1	1	1	1	1	1	23
**Faggella-Luby et al. [59]**	1	1	1	1	0	1	1	1	1	1	1	1	1	1	1	1	1	1	1	1	1	1	1	1	23
**Malone & Mastropieri [38]**	0	1	1	1	1	1	1	0	1	0	1	1	1	1	1	1	1	1	1	1	1	1	1	0	20
**Söğüt & Melekoğlu [67]**	1	1	1	1	0	1	1	1	1	1	1	1	1	1	1	1	1	1	1	1	1	1	1	1	23
**Wong & Jones [33]**	1	1	1	0	0	1	1	0	1	0	1	1	1	1	1	1	1	1	0	1	0	1	1	0	17
**Total**	4	5	4	4	2	5	5	3	5	3	5	5	5	5	5	5	5	5	4	5	4	5	5	3	

*Note*. 1.1 Context/setting description; 2.1 Participant description; 2.2 Participant disability; 3.1 Role and description; 3.2 Training description; 4.1 Intervention procedures; 4.2 Materials description; 5.1 Assessing/reporting implementation fidelity; 5.2 Assessing/reporting fidelity dosage or exposure; 5.3; Assessing fidelity across relevant conditions/throughout study; 6.1 Independent variable (IV) systematically manipulated; 6.2 Control/comparison condition description; 6.3 No or limited access to IV in the control/comparison condition; 6.4 Description of assignment to groups; 6.8 Low overall attrition; 6.9 Low differential attrition; 7.1 Socially important outcomes; 7.2 Description of dependent variable measures; 7.3 Reports effects of the intervention on all measures; 7.4 Appropriate timing of outcome measures; 7.5 Adequate interobserver agreement or reliability; 7.6 Adequate evidence of validity; 8.1 Appropriate data analysis; 8.3 Appropriate effect size reported.

### Student demographics

Across all 17 studies, there were 219 student participants with LD: 199 had only LD (90.9%) and 20 had LD with other disabilities (e.g., ADHD, Health Impairment, Oppositional Defiant Disorder; 9.1%). Male participants were the majority (*n* = 114; 52.1%); there were 45 female participants (20.5%), and gender was not reported for the remaining participants (*n* = 60; 27.4%). Age was reported for 165 students (75.3%) and ranged from 10 to 18 years, with a mean age of 13.6 years. The studies included more middle school students (*n* = 101, 46.1%) than high school students (*n* = 58, 26.5%), but grade level was unclear for 60 students (27.4%). The race of most participants was not reported (*n* = 140; 63.9%). When reported, 42 students (19.2%) were Turkish, 26 students (11.9%) were Caucasian/White, six students (2.7%) were Black/African American, two students (0.9%) were Hispanic, two students (0.9%) were of other races, and one student (0.5%) identified as Biracial (White-Hispanic).

### Implementer roles and settings

Fourteen studies (82.4%) reported one or more intervention implementers, and three studies (17.6%) did not. Among those studies that reported implementers, the interventions were delivered by authors/researchers in eight (57.1%), special education teachers in three (21.4%), general education teachers in four (28.6%), graduate students in two (14.3%), and undergraduate students in one (7.1%). Training was provided to the implementers in only seven of the 17 studies (41.2%). Fourteen studies (82.4%) reported the setting. Of these, six (43%) were conducted in other school settings, three (21.4%) in general education classrooms, three (21.4%) in resource rooms, one (7.1%) in a reading clinic, and one (7.1%) in a special education classroom. For more information, see Table 1.

### Study designs

Twelve studies (70.6%) used single-case designs. Of those studies, eleven (91.7%) employed multiple-probe/baseline designs (including nine multiple-probe/baseline across participants and two multiple-probe/baseline across contexts); one (8.3%) used an alternating treatments design; one (8.3%) used a combination of multiple-probe/baseline designs across participants and a changing criterion design; and one (8.3%) used a combination of multiple-probe/baseline designs across participants and a reversal design. Five studies (29.4%) employed experimental group designs: three were pretest–posttest randomized designs with treatment comparison, one was a posttest randomized design with treatment comparison, and one used a posttest with repeated measures. For more information, see Tables 2 and 3.

### Intervention procedures

Three studies (17.6%) used only one component of SD, whereas fourteen (82.4%) used more than one. Self-monitoring was used in all studies but one (94.1%); self-questioning was used in thirteen studies (76.5%), goal setting and self-instruction were each used in six studies (35.3%), self-reinforcement was used in four studies (23.5%), self-evaluation was used in three studies (17.6%), and multicomponent instructional strategies were used in ten studies (58.8%).

#### Self-monitoring.

Sixteen studies (94.1%) used self-monitoring. Two used it alone, and fourteen combined it with other SD components (self-questioning, *n* = 12; goal setting, *n* = 6; self-instruction, *n* = 6; self-reinforcement, *n* = 4; self-evaluation, *n* = 3). Across the studies, students self-monitored their reading using different methods. Several studies instructed students to use prompt cards and check off strategy steps when applying them [e.g., 18,60,61]. Students were also provided with cue cards that included steps or criteria for performing the target behavior to check while using the strategy [e.g., 33,62]. In addition, students used prompt marks as reminders to stop at predetermined times and answer questions or create questions at the beginning and then answer them at the end [e.g., 19,63]. Students also used checklists while applying the strategy [e.g., 66,67]. Finally, students used a Rocket Chart to monitor their performance in summarizing skills [43].

Other studies had students self-monitor using recording sheets. Students recorded the number of questions completed or answered correctly to calculate percentage of correct responses [68]. Monitoring sheets were developed for students to record their questions and indicate whether they successfully answered them [57] or record their answers during reading [19]. In some studies, students recorded their performance using graphs [18,64,68].

#### Self-questioning.

Across the 13 studies that used self-questioning, only two studies (15.4%) implemented it as a single component of SD interventions: students either were taught to self-generate questions about the text during reading [58] or used questions generated by teachers [59]. In contrast, 12 studies (92.3%) used self-questioning in conjunction with other SD components and/or as a part of a multicomponent instructional strategy. One study, which included three experiments, contributed to both categories because one experiment used self-questioning alone, whereas the other two implemented it in conjunction with other SD components. Therefore, these categories were not mutually exclusive. In eight studies (66.7%), self-questioning was embedded within reading strategies: TWA, TRAP, RAP, IDEAS, SQ3R, ESS, CSI, and summarization training. Students used questions generated by teachers in some of these reading strategies (e.g., TWA [60], TRAP [18]; RAP [62], CSI Group [59], summarization training [38], or students generated questions from headings and subheadings [e.g., 65]. Overall, most of the studies used self-questioning combined with other SD components (self-monitoring, *n* = 12; self-instruction, *n* = 4; goal setting, *n* = 4; self-reinforcement, *n* = 3; and self-evaluation, *n* = 1).

#### Goal setting.

Goal setting was used in six studies and paired with other SD components. For instance, students were taught to set goals to implement the TWA strategy steps at the beginning of their reading [60]. Also, they were taught to set a goal of how much information could be retold when using the TRAP strategy [18].

#### Self-instruction.

Self-instruction was used in six studies and also paired with other SD components. For example, students independently followed and implemented the steps of the strategy [63]. In another study, students were provided with positive self-statements to use and remind themselves during the implementation of the strategy [66].

#### Self-reinforcement.

Self-reinforcement was used in four studies. For example, students were taught to reward themselves for completing the strategy steps by painting a portion of a rocket after each session [60] or by praising themselves and selecting a brief activity to engage in [66].

#### Self-evaluation.

Self-evaluation was used in three studies where students were taught to assess their performance and progress toward their desired goal [e.g., 18,57].

#### Multicomponent instructional strategies.

Ten studies (58.8%) used SD components as part of multicomponent instructional strategies, including TWA, SRSD, TRAP, RAP, SQ3R, ESS, CSI, IDEAS, WINDOW, and the summarization strategy. See Tables 2 and 3 for more information about these intervention procedures.

### Dependent variables

Reading comprehension was the only reading skill targeted across all 17 studies. In some studies, reading comprehension was measured through several dependent measures. Accuracy was measured in 15 studies (88.2%), including the number or percentage of comprehension questions answered correctly, correct identification of main ideas, accurate summaries, and correct paraphrases of key details. Recall accuracy was measured in six studies (35.3%), involving percentages or numbers of pieces of information recalled/retold correctly. Productivity was measured in only one study.

Regarding outcome measures, all but one study measured reading comprehension skills using researcher-developed measures. Only one study employed standardized tests to measure the outcomes [67]. See Tables 2 and 3 for more information about dependent variables.

#### Interobserver agreement and procedural fidelity.

All but three studies (82.4%) reported IOA. The 14 studies that reported it had an average agreement of 80% or more, with a mean IOA of 95.1% across studies (range: 85–100%), and IOA was collected on at least 20% of sessions in 12 studies (85.7%). Eleven studies reported PF (64.7%), with an average PF of 80% or above and a mean PF of 98.9% across studies (range: 95.7–100%); all but one of those studies collected PF data on at least 20% of sessions.

### Social validity

Only eight studies (47.1%) measured social validity: five of these used a survey/questionnaire and three used an interview. Social validity was measured with students in seven studies, compared to only one study that measured it with teachers.

### Maintenance and generalization

Maintenance was assessed in 6 (50%) single-case design studies; however, only 5 of these studies graphed maintenance data. Of those studies, one (16.7%) measured maintenance for up to 2 weeks [65], one (16.7%) measured maintenance for up to 4 weeks [18], one (16.7%) for up to 6 weeks [60], and one (16.7%) for greater than 3 months [61], while two (33.3%) provided an unclear maintenance measure [19,66].

Generalization was measured in only five (29.4%) studies (four single-case and one group design): four studies measured generalization across new materials/texts, and one measured it across new settings. Of the four single-case studies, only two visually graphed the generalization data.

### Intervention intensity

All but two studies (88.2%) included at least one dimension of intervention intensity. Of the 10 single-case studies, seven (70%) reported the number of sessions conducted per day/week (*M* = 3.4; range: 2–5 sessions per week), six (60%) reported the intervention session duration, ranging from 15 to 45 min, and four (40%) reported the total study duration (*M* = 6.8 weeks; range: 4.5 weeks–3 months).

All but one of the group design studies (80%) reported the number of training/instruction days (*M* = 4.25; range: 1–9 days). Session duration and/or total instruction duration were reported in four studies (80%), in which session duration ranged from 45 minutes to 2 hours, and total instruction duration ranged from 1 to 17 hours. Four of the group design studies (80%) reported the total study length (*M* = 6 days; range: 3 days–3 weeks). For more information, see Table 1.

### Efficacy of interventions

For the 12 single-case studies, there were 103 opportunities to show an effect, with 67 demonstrated effects (65%; range: 22.2–100%). The highest percentage of effect was observed on the measure of productivity, demonstrating effects in all three opportunities (100%). A high percentage of effect was also found among accuracy measures, with 43 opportunities for showing an effect and 36 demonstrated effects (83.7%; range: 50–100%). The lowest effect percentage was found among recall accuracy measures, with 26 demonstrations out of 57 opportunities (45.6%; range: 22.2–100%). One study included three experiments and used an alternating treatments design in only one of these experiments, showing 14 separation effects in 30 opportunities (46.7%). Only five studies (41.7%) visually presented maintenance opportunities, showing 15 effects in 19 opportunities (78.9%). Also, only two studies (16.7%) visually presented generalization opportunities and demonstrated three effects in three opportunities (100%).

The group design studies had mixed effects across the outcomes. Malone and Mastropieri [38] found large effects using summarization or summarization with self-monitoring over the control groups on all three recall measures (a posttest, a near-transfer measure of reading texts from the same genre, and a far-transfer measure of reading texts from a different genre), ranging from *d* = 1.35–2.95. When comparing the two treatment groups, the group who received self-monitoring outperformed the summarization group in some recall measures; the effect ranged from no effect on the near-transfer measure (*d* = –0.03) to an approximately large effect on the far-transfer measure (*d* = 0.77). Faggella-Luby et al. [59] found a large effect when using the ESS strategy over the CSI strategy (*d* = 0.93), but Berkeley et al. [57] found no effect on reading accuracy measures when using self-questioning. Söğüt and Melekoğlu [67] found a significantly higher effect for students who received the intervention based on the SDLMI compared to students in the other groups (*d* > 1.82). Finally, we could not calculate the effect size in Wong and Jones’s [33] study because the information needed to calculate it was not reported. However, the results indicated that LD students in the self-questioning training group outperformed their peers in the untrained group in answering comprehension questions, but not in the recall measure. For more details, refer to Tables 2 and 3.

#### Quality of the studies.

Across all the studies, only one single-case design study [58] met all the QIs (22/22 items). For the single-case studies, the QI score range was 13–22, with a mean of 17.5. Out of the 22 items, QI 5.3 (assessing fidelity across relevant conditions/throughout the study) was met in only five studies. Items 4.1, 4.2, and 7.1 were met in all 12 single-case studies.

For the group design studies, no studies met all the QIs. However, three studies (60%) scored 23/24. QI scores for the group studies ranged from 17–23, with a mean of 21.2. QI 3.2 (training description) was met in only two studies. For more information regarding QIs for each study, see Tables 4 and 5.

#### Risk of bias assessment.

Risk-of-bias assessments indicated variation across study designs and domains. Across the 12 single-case design studies, participant selection was assessed as having low risk of bias across all studies, whereas blinding of participants and personnel was judged at high risk in 11 studies, showing some concern. For the five group-design studies, all were assessed as having some concerns overall. Deviations from intended interventions and missing outcome data were at low risk across all five studies, whereas concerns were more common for measurement of the outcome and selection of the reported findings. No group-design study was assessed as having a high risk of bias in any domain. Detailed study-level assessments for all 17 studies are provided in the Supporting information (see S1 Fig and S2 Fig).

## Discussion

The purpose of this paper was to provide a review of studies that examined the effects of SD interventions on reading outcomes for middle and high school students with LD and summarize how components of SD skills were used in those studies. Overall, the findings suggested that SD interventions were generally associated with improvements in reading performance; however, considerable heterogeneity was present across studies in research design, intervention components, implementation procedures, comparison conditions, and outcome measures. This variability limits direct comparisons across studies and requires cautious interpretation of the overall findings.

First, all studies reported at least one element of participants’ demographics (e.g., gender, age, race). However, some demographic data were unclear or not reported in several studies. For instance, gender was reported in 14 studies (82.4%), age was reported in 12 studies (70.6%), and race was reported in only 11 studies (64.7%). Additionally, gender, age, and race were not disaggregated for students with LD in one group design study [57]. Reporting participants’ demographic information is essential because it enables other researchers to replicate the study with similar populations, enhancing the generalizability of the findings [69]. It also aids in identifying potential group differences, such as racial disparities, that may influence the study’s outcomes [70]. Future studies are encouraged to report detailed demographic information to facilitate replication and provide a clear picture of the study populations. In addition, the geographic distribution of the included studies was limited, which may restrict the generalizability of the findings across different educational systems and cultural contexts. Future research should examine SD interventions in more geographically and culturally diverse settings.

Second, we found that certain components of SD were used across all the studies while others received less attention. Specifically, all studies included self-questioning or self-monitoring alone, together, or combined with other SD components or other interventions. This was not surprising, because these components were the focus of several prior studies, including self-monitoring [25,26] and self-questioning [31,71], demonstrating that a high percentage of researchers are interested in implementing self-questioning and self-monitoring to improve reading outcomes. However, limited use of other SD components was noticed. For example, self-reinforcement only appeared in four studies, and self-evaluation was used in only three studies. Despite their importance, self-advocacy and problem solving were not implemented in any of the reviewed studies. Therefore, future research should investigate more SD components and their effects on reading outcomes.

Third, the overall findings indicate that SD interventions positively affected students’ reading outcomes. These findings align with the review by Didion et al. [24], which showed a positive effect of SD interventions on elementary school students with or at risk of LD. However, we found that multiple studies used SD components as part of an intervention package, whether with other SD subcomponents (e.g., goal setting, self-questioning, self-monitoring, and self-reinforcement [60]) or with other reading strategies (e.g., ESS, CSI [59]). Even though the intervention packages demonstrated positive effects, their results should be interpreted cautiously, because it is difficult to determine which SD components actually produced the effects. Examining each component alone can help investigators better understand the effect of that component [72]. We urge future researchers to report and analyze the impact of each SD component individually, as well as examining the effects of using a single SD component versus multiple components.

Fourth, all 17 studies focused only on reading comprehension. This is similar to the finding of Didion et al. [24], who found that the majority of SD studies of elementary students with LD focused on reading comprehension. Based on the current and previous reviews, it is evident that researchers are often interested in examining the effects of SD interventions on reading comprehension as a primary dependent variable. While the primary goal of reading is to comprehend the text [73], other reading skills, such as fluency and vocabulary, are also essential in helping students become good readers, improve their knowledge, and increase their academic success [74,75]. Therefore, future researchers are encouraged to investigate the impact of SD interventions on other reading skills to provide a broader picture of their effects on students with LD.

Another concern is the heavy reliance on researcher-developed outcome measures. All but one study used researcher-developed measures, while only one study employed a standardized measure of reading comprehension. Although researcher-developed measures may be sensitive to intervention-specific changes, their predominance limits comparability across studies and makes it difficult to determine whether observed improvements generalize to broader reading performance. Future studies should therefore incorporate standardized measures alongside intervention-specific assessments.

Fifth, regarding interventions’ efficacy, success estimates across the single-case design studies show that the SD interventions effectively improved reading comprehension in students with LD in middle and high schools. However, as mentioned previously, multiple studies employed intervention packages, which limits the ability to isolate the effects of SD components alone. This makes it challenging to attribute the observed improvements solely to the SD components. There were high success estimates for accuracy (83.7%) and productivity (100%) outcomes. However, recall accuracy, which was examined in four single-case studies, demonstrated a remarkably lower effect (45.6%). This percentage may not generally reflect the overall impact of SD interventions on recall outcome. Specifically, one of these four studies, encompassing three experiments, demonstrated low success estimates of 35.7% across recall outcomes and considerably lowered the overall effect for the recall measure. Excluding this study from the analysis, the overall success estimate for the recall outcome is much higher (83.3%), closely aligning with the other outcome measures. This underscores an essential methodological consideration in systematic reviews of single-case research: a single study with an unusually large or small effect can disproportionately influence overall effects, especially when the total number of studies is limited. More broadly, the evidence base was relatively small, consisting of only 17 studies and 219 participants. This limited evidence base reduces the precision and generalizability of the conclusions regarding the effectiveness of SD interventions.

The effect sizes calculated using Cohen’s *d* across the group-design studies revealed mixed outcomes for SD interventions. However, these effects should be interpreted cautiously because the studies differed in intervention components, comparison conditions, designs, and outcome measures. The single-case studies generally showed a higher proportion of demonstrated effects; however, direct comparisons between the single-case and group-design findings are limited because the two design types were evaluated using different analytic approaches. One potential explanation for the different patterns of findings is that single-case studies often provide more intensive, individualized instruction and repeated opportunities for practice [76]. However, given the methodological differences between the two design types, the current review cannot determine whether these factors account for the differences in observed outcomes.

Sixth, several group design studies were excluded during the full-text reading stage because the results were not disaggregated for LD students in middle or high school. When data are aggregated across participants with and without LD, it is difficult to determine whether the reported outcomes are representative of students with LD. Including such studies would have limited the precision and validity of the conclusions drawn about the effectiveness of interventions for the targeted population [77]. Therefore, to ensure that our findings would be meaningful for practitioners and researchers working with this specific population, only studies that disaggregated outcome data for students with LD were retained. Future researchers should report separate results for students with LD when studying mixed groups. This allows for clearer evaluation of intervention effects and ensures their studies can be included in reviews focused on this population.

Seventh, we found variations in the quality of the studies as measured by the QIs. Only one single-case design study [64] met all the QIs. One limitation of the reviewed studies was that only 11 studies assessed/reported PF (QI 5.1), and only eight assessed PF across conditions or throughout the study (QI 5.3). Assessing PF is crucial because failure to implement procedures consistently may influence students’ outcomes and the overall validity of the study’s findings [78]. Another QI related to PF was training description (QI 3.2). Training intervention implementers is essential to ensure accuracy and fidelity of implementation [79]. However, only seven studies (41.2%) clearly described the training provided to implementers. These methodological weaknesses reduce confidence that the interventions were implemented consistently and make it more difficult to determine whether differences in outcomes across studies reflect intervention effects or variation in implementation. Accordingly, the generally positive findings should be interpreted in light of these limitations. We recommend that future researchers on SD interventions consider assessing/reporting PF and include information on implementer training conducted before the interventions. These elements are critical for ensuring internal validity and also support external validity by improving the clarity, replicability, and generalizability of the findings [46,80].

Another critical QI was socially important outcomes (QI 7.1). Only eight studies (47.1%) reported social validity, which was typically assessed for direct consumers (students) using subjective measures (i.e., questionnaires). Only one study included both direct and indirect consumers (teachers). Research recommends collecting data from other types of stakeholders, such as indirect consumers (e.g., students’ parents and teachers) or immediate community members (e.g., other teachers), as different perspectives can provide a more comprehensive understanding of the intervention’s acceptability among different consumers [79]. Although subjective measures (e.g., questionnaires) are important for measuring the perspectives of stakeholders on the acceptability of an intervention, future research should include objective measures (e.g., direct observation) to investigate whether satisfaction is associated with significant behavior change. Combining both measures can provide a more comprehensive and accurate assessment of social validity [81,82].

Finally, the risk-of-bias assessments identified some methodological concerns across the reviewed studies. Among the single-case design studies, concerns were most frequent for sequence generation and blinding of participants and personnel, whereas participant selection and selective outcome reporting generally showed low risk of bias. For the five group-design studies, all were assessed as having some concerns overall, particularly regarding the measurement of outcomes and selection of reported results. Nevertheless, no study was judged to be at high risk in any domain. Although the reviewed studies demonstrated several methodological strengths, the risk-of-bias results suggest that potential sources of bias should be considered when interpreting the impact of SD interventions. Future researchers should address domains in which concerns were frequently identified, particularly randomization and outcome measurement, to enhance confidence in intervention effects.

### Limitations

This review had some limitations that should be considered. First, we reviewed a small number of studies based on specific criteria. Multiple studies were excluded when they did not directly measure one of the reading skills or when the results for middle or high school students with LD were not disaggregated. Second, we only included studies published in English, which may have excluded findings from SD studies published in other languages.

Third, the review was restricted to studies published in peer-reviewed journals. Although this eligibility criterion was established a priori, it may have increased the potential for publication bias because unpublished studies and gray literature, such as theses and dissertations, were not included. Studies reporting null, smaller, or unfavorable effects may therefore have been underrepresented, which could lead to an overestimation of the consistency or magnitude of intervention effects [83,84]. Formal statistical assessment of publication bias was not conducted because the review did not employ meta-analysis and synthesized heterogeneous research designs using different approaches—success estimates for single-case design studies and Cohen’s *d* for group-design studies—without generating pooled effect estimates and corresponding measures of precision. Therefore, conventional meta-analytic methods for examining publication bias, such as funnel-plot asymmetry tests, were not considered appropriate. Publication bias cannot be ruled out, and the findings should be interpreted with this limitation in mind.

Fourth, the absence of a well-known standard to calculate effect size in single-case designs led us to select a descriptive metric that demonstrated the consistency of the effects in general rather than their magnitude [success estimates; 48]. As empirical evidence continues to develop on effect size metrics, researchers can use different methods in future investigations. Finally, none of the included studies reported or assessed whether participants had previously received instruction in self-determination or similar strategies to improve their reading skills prior to the study. Therefore, determining whether prior exposure to self-determination instruction influenced reading outcomes was not possible. Future researchers are encouraged to report participants’ prior history with self-determination strategies to evaluate whether these factors lead to differential effects.

### Implications for practice

The findings of the reviewed studies suggested that SD interventions were effective in enhancing reading outcomes for middle and high school students with LD. However, given the number of students with LD, implementation of SD interventions in schools is still insufficient [85]. This review has some implications that can guide practice in several ways. First, the SD interventions and materials described in the reviewed studies (sheets, checklists, graphs, prompt cards) were not high-cost or complicated to implement across reading skills. Therefore, instructors can apply different types of SD interventions to teach reading skills more effectively. In addition, it would not take much instructional time to show students how to use them, increasing the opportunity to practice them with different reading tasks.

While the methods used in these studies to teach components of SD were considered easy to implement, professional training for teachers to teach SD skills is still insufficient. A little short training is provided to teachers on SD skills, based on teachers’ reports [86]. This aligns with this review’s findings, in which training was reported in only four studies. Therefore, additional training for teachers to implement SD interventions is needed. Also, teachers can seek out different resources to develop their understanding of teaching SD skills. When SD interventions become common in schools, they could also be applied in peer situations: for instance, high-achieving students, under teachers’ observation and guidance, could teach their LD peers to apply SD skills during reading tasks.

## Conclusion

This review synthesized 17 studies examining SD interventions and reading outcomes for middle and high school students with LD. Across the included studies, SD interventions were generally associated with improvements in reading performance; however, the strength and consistency of these findings should be interpreted cautiously because of substantial variation across studies in research design, intervention components, implementation procedures, and outcome measures. This heterogeneity, together with limited comparability across studies, prevents firm conclusions regarding the overall effectiveness of SD interventions for improving reading outcomes.

All included studies focused on reading comprehension, leaving the effects of SD interventions on other reading skills, such as fluency and vocabulary, largely unknown. Self-monitoring and self-questioning were the most commonly used SD components, whereas other components, including goal setting and self-reinforcement, were used less frequently, and components such as problem-solving and self-advocacy were not represented. In addition, relatively few studies examined maintenance or generalization, and many did not report key participant characteristics.

Although many studies demonstrated methodological strengths, limitations were also evident, including insufficient reporting of procedural fidelity, limited information regarding implementer training, and reliance on subjective measures of social validity. Future research should examine a broader range of SD components and reading outcomes using more comparable and methodologically rigorous procedures. Greater attention is also needed to procedural fidelity, maintenance, generalization, and transparent reporting of participant and intervention characteristics. Such research would allow stronger conclusions regarding when and for whom SD interventions may support reading performance.

## Supporting information

S1 Table Electronic database search strategy and search procedures. (DOCX)

S2 Table List of included and excluded studies with intercoder reliability. (XLSX)

S3 Table List of included studies with coding categories and intercoder reliability. (XLSX)

S1 File PRISMA checklist. (DOCX)

S1 Fig Risk of bias assessment for single-case design studies. (TIF)

S2 Fig Risk of bias assessment for group-design studies. (TIF)
