# Supplementary material for: The effect of self-determination interventions on reading outcomes for middle and high school students with learning disabilities: A systematic review
Source: PLoS One. 2026 Nov 12;21(11):e0362550. doi: 10.1371/journal.pone.0362550 (PMC13759067; doi:10.1371/journal.pone.0362550)
Supplement: Supplementary file 1 [file pone.0362550.s001.docx]

**S1. Table. Electronic database search strategy and search procedures**

| **Database** | **Search platform/interface** | **Search date** | **Search strategy** | **Limits applied** |
| --- | --- | --- | --- | --- |
| APA PsycINFO | The Ohio State University Library search interface | December 2025 | Same complete search strategy shown below | Peer-reviewed; English-language articles |
| ERIC | The Ohio State University Library search interface | December 2025 | Same complete search strategy shown below | Peer-reviewed; English-language articles |
| Academic Search Complete | The Ohio State University Library search interface | December 2025 | Same complete search strategy shown below | Peer-reviewed; English-language articles |
| Academic Search Premier | The Ohio State University Library search interface | December 2025 | Same complete search strategy shown below | Peer-reviewed; English-language articles |
| Education Full Text | The Ohio State University Library search interface | December 2025 | Same complete search strategy shown below | Peer-reviewed; English-language articles |
| Education Research Complete | The Ohio State University Library search interface | December 2025 | Same complete search strategy shown below | Peer-reviewed; English-language articles |

**Search procedure.** The same search strategy, search terms, and limits were applied across all six databases during the December 21st 2025 search. Because the same search strategy was used across databases, a single complete search strategy is provided below rather than reconstructed database-specific search strings.

**Complete Search Strategy**

we developed the following search string of key terms: (“self -determin*” OR “self-monitor*” OR “choice mak*” OR “problem solv*” OR “goal set*” OR “decision mak*” OR “self-regulat*” OR “self-advoc*” OR “self-awar*” OR “self-efficacy” OR “self-instruct*” OR “self-evaluat*” OR “self-reinforce*” OR attain* OR “self-manage*” OR “self-observ*” OR “self-knowledge” OR attribution OR “internal locus of control” OR “self-recruit*” OR “self-question*” OR “self-graph*” OR “self-record*”) AND (“learning dis*” OR “language dis*” OR “special education” OR “disabilit*” OR dyslexi* OR “learning diff*” OR “reading dis*” OR “reading diff*”) AND (reading OR literacy OR phonic* OR “phonemic awareness” OR fluency OR comprehension OR vocabulary) AND (“middle school” OR “junior high” OR “adolescent” OR “high school” OR “secondary education” OR “secondary school” OR “grade 6” OR “grade 7” OR “grade 8” OR “grade 9” OR “grade 10” OR “grade 11” OR “grade 12” OR “6th grade*” OR “7th grade*” OR “8th grade*” OR “9th grade*” OR “10th grade*” OR “11th grade*” OR “12th grade*” OR “sixth grade*” OR “seventh grade*” OR “eighth grade*” OR “ninth grade*” OR “tenth grade*” OR “eleventh grade*” OR “twelfth grade*”).

**Search Results and Duplicate Removal**

The database searches identified 2,203 records. Duplicate records were identified and removed automatically through the library search system before the screening process, resulting in 1,065 unique records for title and abstract screening.

**Citation and record management:**

Duplicate records were automatically identified and removed through The Ohio State University Library search system. The resulting 1,065 unique records were exported to Microsoft Excel for organization and screening.

**Additional Searching**

Following the database search, backward citation searching of the 12 included studies identified one additional article. Forward citation searching was conducted using Google Scholar and identified four additional studies. Hand searching was also conducted in the journals reported in the manuscript.
