# Supplementary material for: The effect of self-determination interventions on reading outcomes for middle and high school students with learning disabilities: A systematic review
Source: PLoS One. 2026 Nov 12;21(11):e0362550. doi: 10.1371/journal.pone.0362550 (PMC13759067; doi:10.1371/journal.pone.0362550)
Supplement: Supplementary file 2 [file pone.0362550.s004.docx]

| **Section and Topic** | **Item #** | **Checklist item** | **Location where item is reported** |
| --- | --- | --- | --- |
| **TITLE** | | |  |
| Title | 1 | Identify the report as a systematic review. | Page 1, Line 1-2 |
| **ABSTRACT** | | |  |
| Abstract | 2 | See the PRISMA 2020 for Abstracts checklist. | Page 2, Line 24-42 |
| **INTRODUCTION** | | |  |
| Rationale | 3 | Describe the rationale for the review in the context of existing knowledge. | Page 4-5, Line 67-100 |
| Objectives | 4 | Provide an explicit statement of the objective(s) or question(s) the review addresses. | Page 5, Line 99- 109 |
| **METHODS** | | |  |
| Eligibility criteria | 5 | Specify the inclusion and exclusion criteria for the review and how studies were grouped for the syntheses. | Page 6, Line 112- 129 |
| Information sources | 6 | Specify all databases, registers, websites, organisations, reference lists and other sources searched or consulted to identify studies. Specify the date when each source was last searched or consulted. | Page 7-8, Line 145- 166 |
| Search strategy | 7 | Present the full search strategies for all databases, registers and websites, including any filters and limits used. | Page 7-8, Line 131- 166 |
| Selection process | 8 | Specify the methods used to decide whether a study met the inclusion criteria of the review, including how many reviewers screened each record and each report retrieved, whether they worked independently, and if applicable, details of automation tools used in the process. | Page 6, Line 112- 129 and Page 16- 17, Line 331- 356 |
| Data collection process | 9 | Specify the methods used to collect data from reports, including how many reviewers collected data from each report, whether they worked independently, any processes for obtaining or confirming data from study investigators, and if applicable, details of automation tools used in the process. | Page 16- 17, Line 331- 356 |
| Data items | 10a | List and define all outcomes for which data were sought. Specify whether all results that were compatible with each outcome domain in each study were sought (e.g. for all measures, time points, analyses), and if not, the methods used to decide which results to collect. | Page 11, Line 235- 245 |
|  | 10b | List and define all other variables for which data were sought (e.g. participant and intervention characteristics, funding sources). Describe any assumptions made about any missing or unclear information. | Page 8-17, Line 168-356 |
| Study risk of bias assessment | 11 | Specify the methods used to assess risk of bias in the included studies, including details of the tool(s) used, how many reviewers assessed each study and whether they worked independently, and if applicable, details of automation tools used in the process. | Page 15, Line 320- 329, Page 16, Line 331- 336 and Page 16, Line 347- 356 |
| Effect measures | 12 | Specify for each outcome the effect measure(s) (e.g. risk ratio, mean difference) used in the synthesis or presentation of results. | Page 13- 14, Line 268- 308 |
| Synthesis methods | 13a | Describe the processes used to decide which studies were eligible for each synthesis (e.g. tabulating the study intervention characteristics and comparing against the planned groups for each synthesis (item #5)). | Page 6, Line 112- 129 |
|  | 13b | Describe any methods required to prepare the data for presentation or synthesis, such as handling of missing summary statistics, or data conversions. | Page 13- 14, Line 268- 308 |
|  | 13c | Describe any methods used to tabulate or visually display results of individual studies and syntheses. | Page 8, Line 161- 163 and Page 17, Line 357- 360 |
|  | 13d | Describe any methods used to synthesize results and provide a rationale for the choice(s). If meta-analysis was performed, describe the model(s), method(s) to identify the presence and extent of statistical heterogeneity, and software package(s) used. | Page 13- 14, Line 268- 308 |
|  | 13e | Describe any methods used to explore possible causes of heterogeneity among study results (e.g. subgroup analysis, meta-regression). | Not applicable |
|  | 13f | Describe any sensitivity analyses conducted to assess robustness of the synthesized results. | Not applicable |
| Reporting bias assessment | 14 | Describe any methods used to assess risk of bias due to missing results in a synthesis (arising from reporting biases). | Not applicable |
| Certainty assessment | 15 | Describe any methods used to assess certainty (or confidence) in the body of evidence for an outcome. | Not applicable |
| **RESULTS** | | |  |
| Study selection | 16a | Describe the results of the search and selection process, from the number of records identified in the search to the number of studies included in the review, ideally using a flow diagram. | Page 7- 8, Line 150- 166 and PRISMA flow diagram in the appendices |
|  | 16b | Cite studies that might appear to meet the inclusion criteria, but which were excluded, and explain why they were excluded. | In the appendices |
| Study characteristics | 17 | Cite each included study and present its characteristics. | Table 1. Implementer, Setting, and Intervention Intensity by Study, Table 2. Designs, Intervention Components, Dependent Variables, and Success Estimates of Single-Case Design Studies, and Table 3. Designs, Intervention Components, Dependent Variables, and Effect Sizes of Group Design Studies |
| Risk of bias in studies | 18 | Present assessments of risk of bias for each included study. | Page 35, Line 544-553 |
| Results of individual studies | 19 | For all outcomes, present, for each study: (a) summary statistics for each group (where appropriate) and (b) an effect estimate and its precision (e.g. confidence/credible interval), ideally using structured tables or plots. | Table 2. Designs, Intervention Components, Dependent Variables, and Success Estimates of Single-Case Design Studies and Table 3. Designs, Intervention Components, Dependent Variables, and Effect Sizes of Group Design Studies |
| Results of syntheses | 20a | For each synthesis, briefly summarise the characteristics and risk of bias among contributing studies. | Page 17- 33, Line 361-505 and Page 34-35, Line 535-553 |
|  | 20b | Present results of all statistical syntheses conducted. If meta-analysis was done, present for each the summary estimate and its precision (e.g. confidence/credible interval) and measures of statistical heterogeneity. If comparing groups, describe the direction of the effect. | Page 33- 34, Line 506-534 and Tables 2–3 |
|  | 20c | Present results of all investigations of possible causes of heterogeneity among study results. | Not applicable |
|  | 20d | Present results of all sensitivity analyses conducted to assess the robustness of the synthesized results. | Not applicable |
| Reporting biases | 21 | Present assessments of risk of bias due to missing results (arising from reporting biases) for each synthesis assessed. | Not applicable |
| Certainty of evidence | 22 | Present assessments of certainty (or confidence) in the body of evidence for each outcome assessed. | Not assessed |
| **DISCUSSION** | | |  |
| Discussion | 23a | Provide a general interpretation of the results in the context of other evidence. | Page 38- 44, Line 554- 693 |
|  | 23b | Discuss any limitations of the evidence included in the review. | Page 44- 45, Line 694-722 |
|  | 23c | Discuss any limitations of the review processes used. | Page 44- 45, Line 694-722 |
|  | 23d | Discuss implications of the results for practice, policy, and future research. | Page 45-46, Line 723-741 |
| **OTHER INFORMATION** | | |  |
| Registration and protocol | 24a | Provide registration information for the review, including register name and registration number, or state that the review was not registered. | This systematic review was not prospectively registered in PROSPERO, the Open Science Framework, the Campbell Collaboration, or another systematic review registry. |
|  | 24b | Indicate where the review protocol can be accessed, or state that a protocol was not prepared. | A formal review protocol was not prepared or registered before the review commenced. Nevertheless, the eligibility criteria, search strategy, screening procedures, data-extraction procedures, and coding framework were established and applied during the review process, and these procedures are reported transparently in the manuscript. |
|  | 24c | Describe and explain any amendments to information provided at registration or in the protocol. | Not available |
| Support | 25 | Describe sources of financial or non-financial support for the review, and the role of the funders or sponsors in the review. | This study is supported by funding from Prince Sattam bin Abdulaziz University under project number PSAU/2026/R/1448. The funder provided support in the form of salaries for authors [M.A.A & A.S.A], but did not have any additional role in the study design, data collection and analysis, decision to publish, or preparation of the manuscript. The specific roles of these authors are articulated in the ‘author contributions’ section. |
| Competing interests | 26 | Declare any competing interests of review authors. | The authors declare that no competing interests exist. |
| Availability of data, code and other materials | 27 | Report which of the following are publicly available and where they can be found: template data collection forms; data extracted from included studies; data used for all analyses; analytic code; any other materials used in the review. | All data are in the manuscript and/or supporting information files. |

*From:*  Page MJ, McKenzie JE, Bossuyt PM, Boutron I, Hoffmann TC, Mulrow CD, et al. The PRISMA 2020 statement: an updated guideline for reporting systematic reviews. BMJ 2021;372:n71. doi: 10.1136/bmj.n71. This work is licensed under CC BY 4.0. To view a copy of this license, visit <https://creativecommons.org/licenses/by/4.0/>
